# Pharmacological basis and clinical evidence of dabigatran therapy

**DOI:** 10.1186/1756-8722-4-53

**Published:** 2011-12-21

**Authors:** Santiago Redondo, Maria-Paz Martínez, Marta Ramajo, Jorge Navarro-Dorado, Abelardo Barez, Teresa Tejerina

**Affiliations:** 1 Service of Hematology, Hospital Nuestra Señora de Sonsoles, Ávila, Spain; 2 Department of Pharmacology, School of Medicine, Universidad Complutense de Madrid, Spain

**Keywords:** dabigatran, thrombosis, surgery, atrial fibrillation

## Abstract

Dabigatran is an emerging oral anticoagulant which is a direct inhibitor of thrombin activity. It has been approved in the European Union and the United States of America for the prevention of thrombosis after major orthopedic surgery. It has also been approved by the American Food and Drug Administration and the European Medicines Agency for the prevention of stroke in chronic atrial fibrillation. Dabigatran provides a stable anticoagulation effect without any need to perform periodical laboratory controls. Of note, there is a growing amount of clinical evidence which shows its safety and efficacy. For these reasons, dabigatran may suppose a revolution in oral anticoagulation. However, two important limitations remain. First, it is contraindicated in patients with end-stage renal disease. Second, there is no evidence of the prevention of thrombosis in mechanical heart valves.

## Introduction

Dabigatran is an emerging drug which acts as a direct and reversible thrombin inhibitor. Due to its predictable pharmacokinetic profile, it is expected to replace, at least in part, vitamin K inhibitors in the prevention of venous thromboembolism and atrial fibrillation. In particular, patients on dabigatran therapy do not need periodic International Normalized Ratio (INR) controls. Studies included in the present review article have been selected from PubMed. For clinical trials, the inclusion criterion was Randomized Clinical Trials. For the search of ongoing trials, the National Institute of Health Registry was consulted http://www.clinicaltrials.gov.

## 1. Pharmacology of dabigatran

### 2.1. Mechanism of action

**Figure 1:**
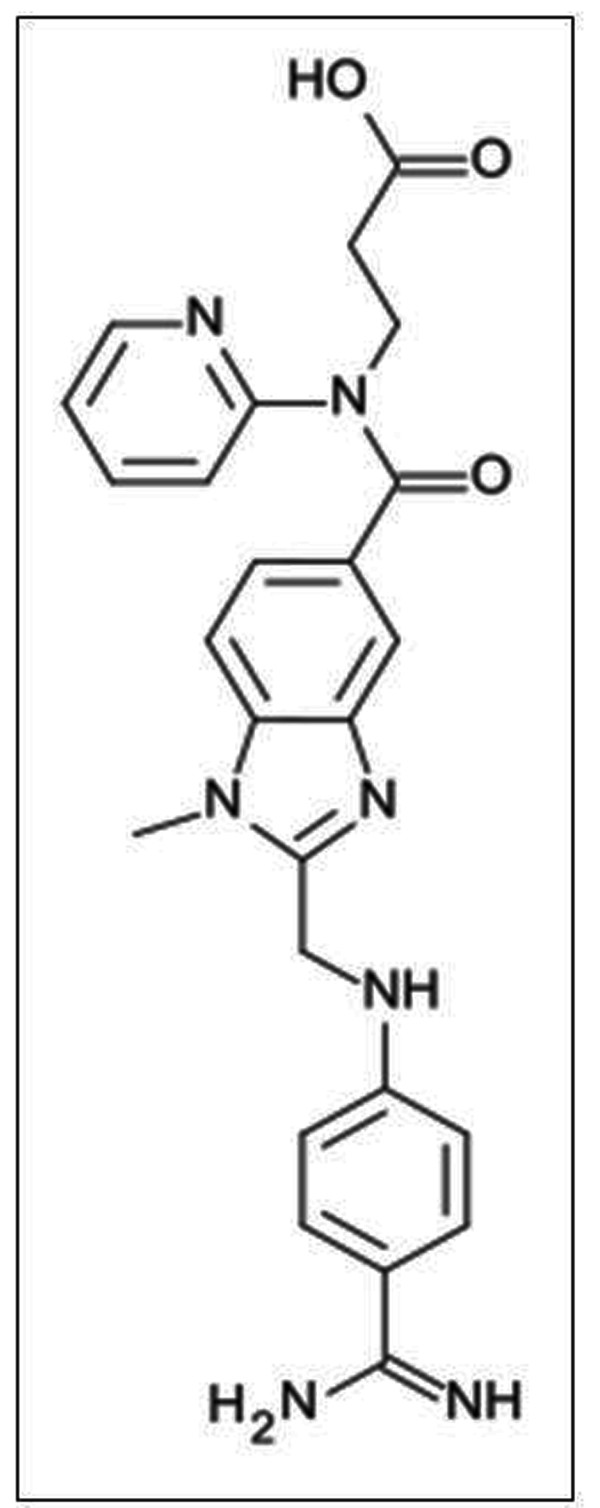
**Chemical structure of dabigatran etexilate or N-[2-[4-[N-(Hexyloxycarbonyl)amidino]phenylaminomethyl]-1-methyl-1H-benzimidazol-5-ylcarbonyl]-N-(2-pyridyl)-beta-alanine ethyl ester**.

The chemical structure of dabigatran is shown in Figure 1. It is a direct inhibitor of thrombin activity (factor II of the human coagulation system). Dabigatran is a peptidomimetic inhibitor of the thrombin. It imitates part of the molecular structure of the fibrinogen, especially in the zone where thrombin and fibrinogen interact and make possible the conversion to fibrin. Dabigatran possesses a benzylimidazolic nucleus, which is bound to a branch of amidinofenylalanine as a false arginine. The dabigatran molecule also possesses a carboxylic residue which increases the hydrophilic capacity of the drug. Thus, dabigatran inhibits the key role of thrombin in human hemostasis. Thrombin plays a central role in the regulation of the coagulation system by activating factors V, XI and fibrinogen itself (factor I).

Dabigatran is a reversible thrombin inhibitor with an inhibition constant of (IC_50_) of 4.5 ± 0.2 nM [1,2]. Dabigatran inhibits the generation of thrombin by platelet-rich plasma in human volunteers [1]. It inhibits platelet aggregation by thrombin with an IC_50 _of 10 mM [1]. However, dabigatran is unable to inhibit the platelet aggregation induced by arachidonic acid, collagen or ADP.

### 2.2. Pharmacokinetics

Pharmacokinetics of dabigatran have been assessed in several studies using repeated doses on human volunteers. Dabigatran is given orally as a prodrug termed dabigatran etexilate. In the peripheral blood, it is activated [3], having a maximum peak time (T_max_) from 0.5 to 1 h, and a maximum concentration (C_max_) of 146 ng/ml, for the dosis of 150 mg twice a day [4]. Its area under curve (AUC) is 1080 ng*h/ml [3]. Dabigatran is bound to the plasmatic proteins in a 35% [1,3] and it is metabolized by plasmatic esterases instead of p450 cytochrome [4].

Distribution volume of dabigatran is 60-70 l, its half-life is 12-24 h and its oral biodisposability is 7% [1,3]. Dabigatran is eliminated in urine and stools. It is mainly excreted in a non-metabolized form by urine by 80%, and conjugated with glucoronic acid and eliminated in stools by 20%. In patients with end-stage renal disease, C_max _of the doses of 150 mg every 12 h can be increased from 100 to 250 ng/ml [5]. Thus, patients with end-stage renal disease were excluded from the clinical trials. However, the current label of the drug in the European Union allows dabigatran for patients with a moderate renal disease (CL_CR _30-50 ml/min), based on pharmacokinetics. In particular, in elderly patients (> 75 years), or those with moderate renal impairment (CL_CR _30-50 ml/min) only the dose of 150 mg is recommended, starting with a half dose [6].

Dabigatran pharmacokinetics is very relevant for its clinical dosing, especially in the post-surgical setting. The BISTRO I study (289 patients) performed a dose-escalating design for the prophylaxis of thrombosis in major orthopedic surgery (hip replacement). In this phase I trial, the first dose was given 4-8 h after surgery. Doses were 12.5, 25, 50, 100, 150, 200 or 300 mg twice a day, and 100 or 300 mg once a day [4]. The primary efficacy endpoint was venous thromboembolism (measured by venography), and the primary safety endpoint was the rate of major bleeding events. The overall rate of thromboembolism was 12.4%, and it decreased from 20.8% at 12.5 mg twice a day to 0% at 300 mg twice a day. No major hemorrhagic events were registered. However, 2 of the 20 patients on 300 mg twice a day developed bleeding from multiple sites. Thus, the optimal therapeutical window was established above 12.5 mg and below 300 mg twice a day [4].

Dabigatran is able to increase the prothrombin time (PT and the ratio INR), activated partial thromboplastin time (APTT), the thrombin time (TT) and the ecarin time (ECT). In clinical trials, APTT was correlated with the blood concentration of the drug. Moreover, there was a close relationship between APTT and the primary efficacy and safety outcomes. Therefore, the higher increase of APTT was associated with decreased thrombosis, increased bleeding, and higher blood concentration of the drug in the two phase II trials BISTRO II (for the prevention of thrombosis in total hip or knee replacement) [7], and PETRO (for the prevention of thromboembolic stroke in chronic atrial fibrillation) [8]. However, the prolongation of the ECT is directly and linearly related to the plasmatic levels of dabigatran. Thus, ECT may be considered as the future test of choice for the clinical monitoring of the effect of dabigatran when needed [5].

### 2.3. Drug interactions

Dabigatran is not metabolized by the p450 cytochrome system. However, the efflux transporter P-gp is involved in the intestinal absortion of dabigatran. This process can be theoretically modulated by P-gp inhibitors (verapamil, quinidine, clarithromycin, ketoconazole and amiodarone), or P-gp inducers (rifampicin). In the practice, the maximum increase in AUC (up to 150%) has been observed with ketoconazole. Quinidine, amiodarone and verapamil can increase bioavailability of dabigatran up to 50% [9]. The current European Medicines Agency label of the drug recommends dose reduction in patients on amiodarone treatment [6].

Antiplatelet drugs may cause bleeding. In atrial fibrillation trials, some patients were treated with dabigatran and aspirin. In the phase II PETRO study, major bleeding events were limited to the group of 300 mg dabigatran twice a day plus aspirin (4 of 64), compared to the group of 300 mg dabigatran twice a day alone (0 of 105, p < 0.02) [8]. However, in the phase III RE-LY trial, aspirin at doses lower than 100 mg was allowed and no significant rate of hemorrhage was observed in the patients who were treated with dabigatran (110 or 150 mg twice a day), plus aspirin [10]. Nevertheless, in this same trial dabigatran 150 mg had significantly more gastrointestinal bleeding as compared to coumadin (182 v/s 120 patients, p < 0.001) [10].

### 2.4. Secondary effects

Given that hepatotoxicity was the reason for the clinical withdrawal of ximelagatran [11], the previous direct thrombin inhibitor, the hepatic biochemical function has been intensively studied in the clinical trials of dabigatran.

To date, not a single clinical trial has demonstrated a significant increase of transaminase enzymes. In dose-escalating trials, a dose-dependent increase of transaminase enzymes is equally not observed [4,5,10,12].

A common adverse effect of dabigatran is dyspepsia. This may be due to the pills being embedded in tartaric acid to facilitate its absorption [9].

Dabigatran increased urinary excretion of 11-dehydrothromboxane B2 (DTB2) up to 20% in the phase II PETRO study [8]. This could theoretically increase the thrombotic risk. Of note, this DTB2 increase was not observed when dabigatran was given with aspirin in this clinical trial [8]. Ximelagatran, the previous thrombin inhibitor, decreased cardiac events when given in addition to aspirin [11].

## 3. Clinical evidence

### 3.1. Dabigatran for total hip or knee replacement surgery

Prophylaxis of thrombosis in hip and knee replacement surgery is the first indication approved for dabigatran, by the European Medicines Agency (EMA) and the United States Food and Drug Administration (FDA). The BISTRO I study, which has been commented on above, was a multicentric and sequential phase I trial where dabigatran was used in an escalating-dose schedule on 289 patients [4]. Results showed a reasonable therapeutic window for the drug, with low risks of thrombosis and hemorrhage, in patients over 12.5 mg once a day, and below 300 mg twice a day [4]. The BISTRO II was a double-blind phase II trial to compare the efficacy and safety of dabigatran versus daily enoxaparin (40 mg a day) on 1973 patients [7]. The primary efficacy outcome was the rate of venous thrombosis (measured by venogram), whereas the primary safety endpoint was the risk of major bleeding. Dabigatran showed a clear dose-dependent antithrombotic effect (p < 0.0001). Compared with enoxaparin 40 mg once a day, dabigatran achieved a lower rate of thrombotic events, for the doses of 150 mg twice a day (OR 0.65, p = 0.04), 300 mg once a day (OR 0.61, p = 0.02), and 225 mg twice a day (OR 0.47, p = 0.0007). Compared to the enoxaparin group, dabigatran showed a lower hemorrhagic risk for the dose of 50 mg twice a day (0.3% v 2%, p = 0.047), though the difference was not significant for the doses of 150 mg twice a day (4.1%, p = 0.1), 225 mg twice a day (3.8%, p = 0.15), and 300 mg once a day (4.7%, p = 0.051) [7].

The RE-MODEL was a randomized, prospective, double-blind, non-inferiority phase III trial which was designed to compare dabigatran and enoxaparin 40 mg once a day on 2076 patients who underwent knee replacement surgery [12]. Dabigatran was used at a dose of 150 mg or 220 mg once a day. The first dose was one-half of subsequent doses and it was administered 1-4 h after completion of the surgery. Compared to enoxaparin, dabigatran did not show a different profile in terms of prevention of thrombosis and hemorrhage. The primary efficacy outcome was a composite endpoint of venographic thrombosis and/or clinical thrombosis and mortality. This efficacy outcome had a rate of 37.7% in the enoxaparin group vs. 36.4% in the dabigatran 220 mg/24 h group (95% CI -7.3 to 4.6), and 40.5% in the 150 mg/24 h group (95% to CI -3.1 to 8.7). The rate of major hemorrhage was 1.3% in the enoxaparin group, 1.5% in dabigatran 220 mg (p = 0.82) and 1.3% in dabigatran 150 mg (p = 1.0) [12].

Similar conclusions were achieved by the RE-NOVATE trial for hip replacement [13]. It was a prospective, phase III, randomized clinical trial on 3494 patients. The primary efficacy endpoint was the composite of total thromboembolic events and all-cause mortality, while the safety outcome was the rate of major hemorrhage. Enoxaparin 40 mg once a day had a rate of 6.7% of thrombosis/mortality. Dabigatran showed a non-inferior efficacy profile for both doses (6% in 220 mg once a day, p < 0.0001 for non-inferiority, and 8.6% for 150 mg one a day, p < 0.0001 for non-inferiority). The rate of major bleeding was also not significant for dabigatran compared to enoxaparin 40 mg once a day. In particular, the enoxaparin group had a hemorrhagic risk of 1.6%, while dabigatran 220 mg had a 1% (p = 0.44), and dabigatran 150 mg had 1.3% (p = 0.60) [13].

The double-blind RE-MOBILIZE trial, however, reached different results [14]. It was a prospective, double-blind, randomized phase III trial for the prevention of thrombosis after knee arthroplasty on 2596 patients. In this study, enoxaparin was used at 30 mg twice a day (the American standard) vs dabigatran at 220 or 150 mg once a day [14]. The primary efficacy and safety outcomes were the same as described for RE-MODEL and RE-NOVATE. Dabigatran failed to show a non-inferior efficacy compared to enoxaparin 30 mg twice a day. The risk difference was 5.8% for dabigatran 220 mg (95% CI, 0.8-10.8, p = 0.0234) and 8.4% for dabigatran 150 mg (95% CI, 3.4-13.3, p = 0.0009). The rate of bleeding events, however, was not statistically significant among groups [14].

A meta-analysis performed a pooled analysis of these three trials: RE-MODEL, RE-NOVATE and RE-MOBILIZE (8135 patients) [15]. The pooled rate of thrombosis or thrombosis-related mortality was 3.3% in the enoxaparin group vs 3% of dabigatran 220 mg group (p = 0.20), and 3.8% of the dabigatran 150 mg group (p = 0.91). The rate of major bleeding was 1.4% in the enoxaparin group vs. 1.4% in dabigatran 220 mg (p = 0.61) and 1.1% in dabigatran 150 mg (p = 0.16). Subgroup analyses suggested that bleeding risk appeared to be higher in patients with moderate renal impairment (Cl_CR _30-50 ml/min) and in patients older than 75 years. There was no evidence of statistical heterogeneity among the three trials [15].

Thus, the current clinical evidence strongly supports the indication of dabigatran for the prevention of thrombosis in patients who underwent hip or knee replacement surgery, with a reasonable efficacy and safety profile. Dabigatran has been approved for this indication in the European Union for the dose of 220 mg once a day, with a starting dose of 110 mg 1-4 h after surgery. For patients with moderate renal impairment, older than 75 years, or on amiodarone treatment, the dose of 75 mg twice a day is recommended (with a starting dose of 75 mg 1-4 h after surgery) [6].

### 3.2. Other situations of deep venous thrombosis/pulmonary embolism

The RE-COVER study was a randomized, double-blind and non-inferiority phase III trial where dabigatran and warfarin were evaluated for the treatment of deep venous thrombosis on 2564 patients [16]. Follow-up was 6 months and low molecular weight heparin was used prior to oral anticoagulation in both groups. Dabigatran was administered at a constant dose of 150 mg twice a day, whereas warfarin was used at a dose to achieve an INR between 2 and 3. The efficacy endpoint was a composite of symptomatic venous thromboembolism or related death. It had a rate of 2.4% in the dabigatran group and 2.1% in the warfarin group (hazard ratio 1.10, 95% CI, 0.65-1.84). Dabigatran proved non-inferior with regards to the prevention of recurrent or fatal venous thromboembolism (p < 0.001 for non-inferiority). Of note, the rate major or clinical relevant non-major bleeding events was 5.6% in the dabigatran group vs. 8.8% in the warfarin group (hazard ratio 0.63%, 95% CI, 0.47-0.84, p = 0.0002 [16]. Therefore, dabigatran may suppose a future clinical choice for the treatment of deep venous thrombosis/pulmonary embolism.

### 3.2. Dabigatran for chronic atrial fibrillation

The PETRO randomized phase II trial compared the effect of vitamin K antagonists versus dabigatran at doses at 50, 150 or 300 mg twice a day, with or without aspirin at 82 or 325 mg, on 502 patients [8]. The primary outcome was the rate of bleeding events. Major bleeding events were limited to the group treated with 300 mg dabigatran twice a day plus aspirin compared to 300 mg dabigatran twice a day alone (p < 0.02). Total bleeding events were lower in the 50 mg group (7%), compared with the 300 mg group (23%, p = 0.0002) and the 150 group (18%, p = 0.01). The group of 50 mg had a rate of 2%of thromboembolic events. Therefore, an optimal dose from 150 mg twice a day to lower than 300 mg twice a day was selected for further studies [8].

The randomized, double-blind RE-LY phase III trial assessed the efficacy and safety profile of dabigatran (110 or 150 mg twice a day) on 18113 patients, compared to warfarin. The median duration of the follow-up period was 2 years. The primary efficacy outcome was stroke or systemic embolism. The primary safety outcome was a major hemorrhage [10]. Stroke or systemic embolism had a rate of 1.53% per year in the group of dabigatran 110 mg twice a day, and 1.11% per year in the group of dabigatran 150 mg twice a day. Both doses of dabigatran were non-inferior to warfarin (p < 0.001). For the prevention of thrombotic events, the dose of 150 mg was superior to warfarin (relative risk 0.66, 95% CI, 0.53-0.82, p < 0.001), whereas the dose of 110 mg was not (relative risk 0.91, 95% CI, 0.74-1.11, p = 0.34). In contrast, the rate of major bleeding was 3.36% per year in the warfarin group, while it was 3.11% per year in the 150 mg dabigatran group (relative risk of 0.93, CI 95%, 0.81-1.07, p = 0.31) and 2.71% per year in the 110 mg dabigatran group (relative risk of 0.8, CI 95%, 0.69-0.93, p = 0.003) [10].

The FDA approved dabigatran for the prevention of thrombosis in atrial fibrillation in October, 2010. Recommended doses are 150 mg twice a day for Cl_CR _> 30 ml/min, and 75 mg twice a day for patients with moderate renal impairment (15-30 ml/min) [17]. This indication has also recently been approved by the EMA [6].

### 3.3. Dabigatran for mechanical heart prosthesis

To date, we still do not have any clinical evidence to evaluate the effect of dabigatran for the prophylaxis of thrombosis in this clinical setting. No ongoing clinical trials have been found in http://www.clinicaltrials.gov.

## 4. Bleeding complications in patients on dabigatran

Despite the favorable safety profile for dabigatran, several concerns exist regarding the best medical care for patients on dabigatran who suffer from overdosage and bleeding complications (for example in acute renal failure), and patients on need of urgent surgery or other invasive procedures, given the lack of antidote for dabigatran. If possible, the most effective and safe procedure is discontinuation of the drug, which should be discontinued at least 24 h prior to surgery [18]. However there is still a lack of clinical evidence about dabigantran antagonism in an emergent clinical setting. The 2011 ACCF/AHA guidelines suggest the usage of fresh-frozen plasma, although no clinical trials have been conducted [19]. This intervention has been questioned from a theoretical point of view, given that plasma contains prothrombin. However, it is widely regarded as an efficient procedure to restore clotting factor depletion, but not a clotting factor inhibition [20]. The usage of prothrombin complex may also be theoretically advocated. However, data from healthy volunteers (n = 12) show that prothrombin complex is unable to normalize APTT, TT and ECT [21]. Nevertheless, activated prothrombin complex (Feiba^® ^50 to100 U/kg) reversed bleeding time, but not APTT, in a rat model of dabigatran overdose [18], although data from humans are still lacking. Recombinant factor VII (90 mg/kg) has equally been advocated, although small human studies in melagatran (not in dabigatran) have yielded inconsistent findings [18]. To date, the only proven procedure which has been demonstrated to rapidly decrease plasma concentration of dabigatran is haemodyalisis. Data from an open-label pharmacokinetic study in patients with end-stage renal failure (n = 6, dose of 50 mg) show that the mean fraction of the drug removed was 62-68% at 2 h, with a parallel decrease of APTT and ECT [22]. Yet again, no clinical data about the role of haemodyalisis in dabigatran bleeding complications or antagonism for emergency surgery are available. The question arises about how to monitor bleeding complications of dabigatran when TT and ECT are lacking. Pharmacokinetic data suggest that, in this case, APTT may be the most useful test for a qualitative monitoring of dabigatran [18].

## 5. Conclusion: Advantages and disadvantages of dabigatran

Dabigatran may suppose a significant change in oral anticoagulation with a reasonable efficacy and safety profile, based on the current clinical evidence (Table 1). This drug may become the oral anticoagulant of choice in many clinical situations, after an individualized evaluation of advantages and disadvantages in every single patient.

**Table 1:** Clinical studies with dabigatran etexilate

Study	Phase	N	Indication	Efficacy	Safety
Bistro I [4]	I	289	HR or KR	20.8% VTE in 12.5 mg/12 h vs. 0% 300 mg/12 h	10% bleeding in 300 mg/12 h

Bistro II [7]	II	1973	HR or KR	Lower VTE in 150 mg/12 h, 225 mg/12, 300 mg/24 h, compared to enoxaparin 40 mg/24 h	Lower bleeding than heparin in 50 mg/12 h

RE-MODEL [12]	III	2076	KR	Similar for 150 and 220 mg/24 h compared to enoxaparin 40 mg/24 h	Similar bleeding rate

RE-NOVATE [13]	III	3463	HR	Similar efficacy among the same groups	Similar bleeding rate

RE-MOBILIZE [14]	III	2596	KR	Lower VTE in enoxaparin 30 mg/12 h compared to dabigatran 150 and 220 mg	Similar bleeding rate

Friedman et al., [15]	MA	8135	HR and KR	Similar VTE risk among groups (3 trials)	Similar bleeding rate

RE-COVER [16]	III	2564	DVT/PE	Similar efficacy for dabigatran 150 mg/12 h compared to warfarin	Lower bleeding in the dabigatran group

PETRO [8]	II	502	AF	2% thromboembolic events when the lowest dose was used (50 mg/12 h)	Lower bleeding in 50 mg than 150 mg and 300 mg (twice a day)

RE-LY [10]	III	18113	AF	Dose of 150 mg/12 h had lower thromboembolic events than warfarin. No difference for 110 mg/12 h	Lower major bleeding rate for the dose of 110 mg/12 h, compared to warfarin.

Efficacy and safety headings only describe the conclusions. A deeper description of each study is made in the Text. VTE: Venous thromboembolism. AF: Atrial fibrillation. HR: Hip replacement. KR: Knee replacement. MA: Meta-analysis. DVT/PE: Deep vein thrombosis/pulmonary embolism.

The major advantages of dabigatran over vitamin K antagonists (VKA) are: the absence of periodic laboratory analysis, the low extent of dietary and drug interactions and the favorable safety-efficacy profile, which may decrease the rate of clinical complications due to an over abundance of vitamin K inhibitors in selected patients. The major disadvantages, in addition to the high prize of the drug, are the lack of evidence in mechanical heart valves, the dependence of a proper renal function, and the lack of experience in dabigatran-associated hemorrhage and reversion for emergent invasive procedures (Table 2).

**Table 2:** Advantages and disadvantages of dabigatran over VKA

Advantages over VKA	Disadvantages over VKA
No need of periodic INR control	High prize

Lower interactions	Not indicated in renal disease

Favorable safety versus efficacy profile, it may reduce VKA misdosing	No antidote and lack of experience in hemorrhage complications

In conclusion, day-to-day experience in the clinical arena and high-level clinical evidence will eventually set the enormous potential importance of this drug in oral anticoagulation and enhance their safety profile. Clinicians should be aware of the coming of this putative revolutionary change in the field of oral anticoagulation.

## Abbreviations

INR: International Normalized Ratio; IC_50_: half-maximal inhibitory concentration; T_max_: Time for peak concentration; C_max_; Peak concentration; Cl_CR_: Creatinine clearance; PT: Prothrombin time; APTT: Activated partial thromboplastin time; TT: Thrombin time; ECT: Ecarin clotting time; AUC: Area under curve; EMA: European Medicines Agency; FDA: Food and Drug Administration; OR: Odds ratio; CI: Confidence interval; DTB2: 11-dehydrothromboxane B2; VTE: Venous thromboembolism; AF: Atrial fibrillation; HR: Hip replacement; KR: Knee replacement; MA: Meta-analysis; DVT/PE: Deep vein thrombosis/pulmonary embolism; VKA: Vitamin K antagonists.

## Competing interests

The authors declare that they have no competing interests.

## Authors' contributions

SR and TT developed the original idea of the manuscript and undertook the first bibliographic research. MR, JN-D, MPM and AB participated in the final draft of the work. All authors read and approved the final manuscript.

## References

[B1] Wienen W Stassen JM Priepke H In-vitro profile and ex-vivo anticoagulant activity of the direct thrombin inhibitor dabigatran and its orally active prodrug, dabigatran etexilate Thromb Haemost 2007 98 155 162 17598008

[B2] Garcia D Libby E Crowther MA The new oral anticoagulants Blood 2010 115 15 20 10.1182/blood-2009-09-241851 19880491

[B3] Stangier J Clinical pharmacokinetics and pharmacodynamics of the oral direct thrombin inhibitor dabigatran etexilate Clinical Pharmacokinetis 2008 47 285 295 10.2165/00003088-200847050-00001 18399711

[B4] Eriksson BI Dahl OE Ahnfelt L Kälebo P Stangier J Nehmiz G Hermansson K Kohlbrenner V Dose escalating safety study of a new oral direct thrombin inhibitor, dabigatran etexilate, in patients undergoing total hip replacement: BISTRO I J Thromb Haemost 2004 2 1573 1580 10.1111/j.1538-7836.2004.00890.x 15333033

[B5] Stangier J Rathgen K Stähle H Gansser D Roth W The pharmacokinetics, pharmacodynamics and tolerability of dabigatran etexilate, a new oral direct thrombin inhibitor, in healthy male subjects Br J Clin Pharm 2007 64 292 303 10.1111/j.1365-2125.2007.02899.x PMC2000643 17506785

[B6] European Medicines Agency Dabigatran label http://www.ema.europa.eu

[B7] Eriksson BI Dahl OE Büller HR Hettiarachchi R Rosencher N Bravo ML Ahnfelt L Piovella F Stangier J Kälebo P Reilly P BISTRO II Study Group A new oral direct thrombin inhibitor, dabigatran etexilate, compared with enoxaparin for prevention of thromboembolic events following total hip or knee replacement: the BISTRO II randomized trial J Thromb Haemost 2005 3 103 111 10.1111/j.1538-7836.2004.01100.x 15634273

[B8] Ezekowitz MD Reilly PA Nehmiz G Simmers TA Nagarakanti R Parcham-Azad K Pedersen KE Lionetti DA Stangier J Wallentin L Dabigatran with or without concomitant aspirin compared with warfarin alone in patients with nonvalvular atrial fibrillation (PETRO Study) Am J Cardiol 2007 100 1419 1426 10.1016/j.amjcard.2007.06.034 17950801

[B9] Boerhinger-Ingelheim Dabigatran Advisory Committee Briefing Document http://www.fda.org

[B10] Connolly SJ Ezekowitz MD Yusuf S Eikelboom J Oldgren J Parekh A Pogue J Reilly PA Themeles E Varrone J Wang S Alings M Xavier D Zhu J Diaz R Lewis BS Darius H Diener HC Joyner CD Wallentin L RE-LY Steering Committee and Investigators Dabigatran versus warfarin in patients with atrial fibrillation N Engl J Med 2009 361 1139 1151 10.1056/NEJMoa0905561 19717844

[B11] Wallentin L Wilcox RG Weaver WD Emanuelsson H Goodvin A Nyström P Bylock A ESTEEM Investigators Oral ximelagatran for secondary prophylaxis after myocardial infarction: the ESTEEM randomised controlled trial Lancet 2003 362 789 797 10.1016/S0140-6736(03)14287-0 13678873

[B12] Eriksson BI Dahl OE Rosencher N Kurth AA van Dijk CN Frostick SP Kälebo P Christiansen AV Hantel S Hettiarachchi R Schnee J Büller HR RE-MODEL Study Group Oral dabigatran etexilate vs. subcutaneous enoxaparin for the prevention of venous thromboembolism after total knee replacement: the RE-MODEL randomized trial J Thromb Haemost 2007 5 2178 2185 10.1111/j.1538-7836.2007.02748.x 17764540

[B13] Eriksson BI Dahl OE Rosencher N Kurth AA van Dijk CN Frostick SP Prins MH Hettiarachchi R Hantel S Schnee J Büller HR RE-NOVATE Study Group Dabigatran etexilate versus enoxaparin for prevention of venous thromboembolism after total hip replacement: a randomised, double-blind, non-inferiority trial Lancet 2007 370 949 956 10.1016/S0140-6736(07)61445-7 17869635

[B14] Ginsberg JS Davidson BL Comp PC RE-MOBILIZE Writing Committee RE-MOBILIZE Writing Committee Ginsberg JS Davidson BL Comp PC Francis CW Friedman RJ Huo MH Lieberman JR Muntz JE Raskob GE Clements ML Hantel S Schnee JM Caprini JA Oral thrombin inhibitor dabigatran etexilate vs North American enoxaparin regimen for prevention of venous thromboembolism after knee arthroplasty surgery J Arthroplasty 2009 24 1 9 18534438 10.1016/j.arth.2008.01.132

[B15] Friedman RJ Dahl OE Rosencher N Caprini JA Kurth AA Francis CW Clemens A Hantel S Schnee JM Eriksson BI RE-MOBILIZE RE-NOVATE Steering Committees Dabigatran versus enoxaparin for prevention of venous thromboembolism after hip or knee arthroplasty: A pooled analysis of three trials J Thromb Haemost 2010 126 175 182 10.1016/j.thromres.2010.03.021 20434759

[B16] Schulman S Kearon C Kakkar AK Mismetti P Schellong S Eriksson H Baanstra D Schnee J Goldhaber SZ RE-COVER Study Group Dabigatran versus Warfarin in the Treatment of Acute Venous Thromboembolism New Engl J Med 2009 361 2342 2352 10.1056/NEJMoa0906598 19966341

[B17] Food and Drug Administration Dabigatran label http://www.fda.org

[B18] Van Ryn J Stangier J Haertter S Liesenfeld KH Wienen W Feuring M Clemens A Dabigatran etexilate--a novel, reversible, oral direct thrombin inhibitor: interpretation of coagulation assays and reversal of anticoagulant activity Thromb Haemost 2010 103 1116 1127 10.1160/TH09-11-0758 20352166

[B19] Wann LS Curtis AB Ellenbogen KA Estes NA Ezekowitz MD Jackman WM January CT Lowe JE Page RL Slotwiner DJ Stevenson WG Tracy CM 2011 ACCF/AHA/HRS focused update on the management of patients with atrial fibrillation (update on dabigatran): a report of the American College of Cardiology Foundation/American Heart Association Task Force on practice guidelines J Am Coll Cardiol 2011 57 1330 1337 10.1016/j.jacc.2011.01.010 21324629

[B20] Ganetsky M Babu KM Salhanick SD Brown RS Boyer EW Dabigatran: Review of Pharmacology and Management of Bleeding Complications of This Novel Oral Anticoagulant J Med Toxicol 2011 in press 10.1007/s13181-011-0178-y PMC3550194 21887485

[B21] Eerenberg ES Kamphuisen PW Sijpkens MK Meijers JC Buller HR Levi M Reversal of rivaroxaban and dabigatran by prothrombin complex concentrate: a randomized, placebo-controlled, crossover study in healthy subjects Circulation 2011 124 1573 9 10.1161/CIRCULATIONAHA.111.029017 21900088

[B22] Stangier J Rathgen K Stähle H Mazur D Influence of renal impairment on the pharmacokinetics and pharmacodynamics of oral dabigatran etexilate: an open-label, parallel-group, single-centre study Clin Pharmacokinet 2010 49 259 68 10.2165/11318170-000000000-00000 20214409

